# Clinical decision support automates care gap detection among primary care patients with nonalcoholic fatty liver disease

**DOI:** 10.1097/HC9.0000000000000035

**Published:** 2023-02-09

**Authors:** Ashley Spann, Kristy M. Bishop, Asli O. Weitkamp, Shane P. Stenner, Scott D. Nelson, Manhal Izzy

**Affiliations:** 1 Division of Gastroenterology, Hepatology, and Nutrition, Vanderbilt University Medical Center, Nashville, Tennessee, USA; 2 Department of HealthIT, Vanderbilt University Medical Center, Nashville, Tennessee, USA; 3 Department of Biomedical Informatics, Vanderbilt University Medical Center, Nashville, Tennessee, USA

## Abstract

Although guidelines recommend primary care–driven management of NAFLD, workflow constraints hinder feasibility. Leveraging electronic health records to risk stratify patients proposes a scalable, workflow-integrated strategy.

**Materials and Methods:**

We prospectively evaluated an electronic health record-embedded clinical decision support system’s ability to risk stratify patients with NAFLD and detect gaps in care. Patients missing annual laboratory testing to calculate Fibrosis-4 Score (FIB-4) or those missing necessary linkage to further care were considered to have a gap in care. Linkage to care was defined as either referral for elastography-based testing or for consultation in hepatology clinic depending on clinical and biochemical characteristics.

**Results:**

Patients with NAFLD often lacked annual screening labs within primary care settings (1129/2154; 52%). Linkage to care was low in all categories, with <3% of patients with abnormal FIB-4 undergoing further evaluation.

**Discussion:**

Significant care gaps exist within primary care for screening and risk stratification of patients with NAFLD and can be efficiently addressed using electronic health record functionality.

## Graphical abstract

**Figure:**
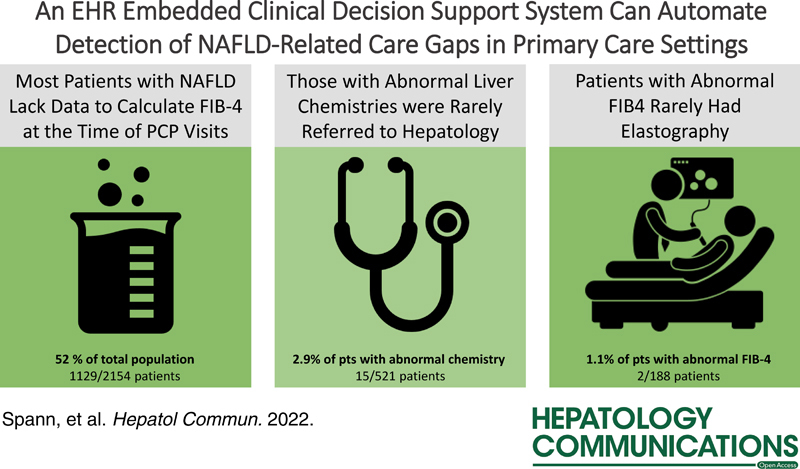

## INTRODUCTION

NAFLD is highly prevalent worldwide, ranging from steatosis to steatohepatitis (NASH) and cirrhosis, which can lead to HCC.^1^ To this end, NASH has become a common indication for liver transplantation.^2^ Unfortunately, patients with NAFLD are often either unaware of it or are identified late.^3^,^4^ Despite the lack of approved pharmacotherapy for NAFLD, early weight-based intervention reverses the disease and HCC screening in advanced fibrosis or cirrhosis improves survival.^5^ However, opportunities for early interventions are frequently missed.^6^

Recent guidelines recommended NAFLD assessment within primary care using noninvasive testing tools like Fibrosis-4 Score (FIB-4).^7^ However, screening can be difficult to routinely perform in an already time-constrained clinic, if not embedded seamlessly within providers workflows.^8^ Therefore, electronic health record (EHR)–based tools may allow for automated, consistent identification and risk stratification of patients with NAFLD. Thus, we sought to provide a proof-of-concept study for leveraging EHR functionality for care gap identification and assessing linkage rates to NAFLD-related care.

## MATERIALS AND METHODS

**FIGURE 1:**
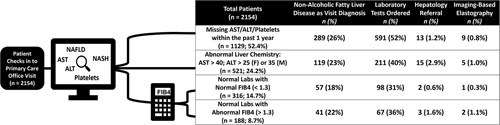
Patient categorization and associated linkage to care through addressing NAFLD during the visit, screening laboratory assessment, hepatology referral, or imaging-based Elastography. AST, aspartate transaminase; ALT, alanine transaminase; FIB-4, Fibrosis-4 Score; NAFLD, nonalcoholic fatty liver disease; NASH, nonalcoholic steatohepatitis.

Our center uses Epic (Epic Systems, Verona, WI) as its EHR. Using native EHR functionality, we created a clinical decision support system to be triggered during primary care visits for qualifying patients as identified by documented diagnostic codes (Figure 1). Patients were included if they had a primary care provider within our institution and had not visited a hepatologist within 3 years. By these qualifications, we prospectively tracked 2154 patients seen during primary care in office or telemedicine visits over a 4-month period (November 16, 2021, to March 16, 2022) for gaps in care (Table A1). Gaps in care were identified in patients missing annual internal or external laboratory testing to calculate FIB-4 or missing linkage to further care, when indicated. Linkage to care was defined as referral for elastography-based testing or for consultation in hepatology clinic depending on clinical and biochemical characteristics. The study was approved by the Institutional Review Board.

**TABLE A1:** Patient clinical characteristics

Demographic information	Patients (n=2154)
Age at visit	60 (49, 69)
History of hypertension	1435 (67)
History of type 2 diabetes	871 (40)
NAFLD diagnosis addressed during visit	506 (23.5)
Sex	
Male	990 (46)
Female	1164 (54)
Visit type	
Office visit	1951 (91)
Telemedicine	203 (9.4)
Race	
White	1771 (82)
Black	182 (8.4)
Asian	60 (2.8)
Other/not reported	141 (6.5)
Ethnicity	
Hispanic or Latino	90 (4.2)
Non-Hispanic or non-Latino	1935 (90)
Not reported	129 (6)
Clinical data review	
Systolic blood pressure (n=1942)	128 (119, 138)
Diastolic blood pressure (n=1942)	76 (68, 83)
Body mass index (n=1957)	33 (29, 38)
Hemoglobin A1c (n=1224)	6.4 (5.7, 7.6)
Aspartate aminotransferase (n=1549)	26 (21, 37)
Alanine aminotransferase (n=1559)	29 (20, 46)
Platelets (n=1443)	253 (204, 301)
Low-density lipoprotein (n=1249)	91 (67, 117)
High-density lipoprotein (n=1246)	45 (38, 54)
Triglycerides (n=1249)	145 (104, 206)

Continuous variables reported as median (IQR); categorical variables reported as n (%).

Once patients were included, they were categorized based on their existing EHR data at the time of the visit (Figure 1). The 4 categories were missing screening labs (no aspartate transaminase, alanine transaminase, or platelet count within the past 365 days), abnormal liver chemistry (aspartate transaminase >40 or elevated alanine transaminase (>25 females, >35 males per society guidelines), and normal labs (normal aspartate transaminase/alanine transaminase level) with either normal FIB-4 (<1.3) or abnormal FIB-4 (>1.3). Patients with abnormal liver enzymes were separately categorized given varied guideline recommendations for subsequent management.^7^,^9^

## RESULTS

Despite the established NAFLD diagnoses, most patients were missing screening labs within the past year (1129/2154; 52%). The second largest category was patients with abnormal liver chemistry, accounting for 24% of the total patient cohort (521/2154, 24%). 44% of these patients (229/521) had an abnormal FIB-4. Among 504 patients with normal liver chemistry, 316 had a normal FIB-4 (15% of total cohort), whereas 188 had abnormal FIB-4 (9% of total cohort).

Although representing the highest group of referrals, only 2.9% of patients with abnormal liver chemistries were referred. For patients with elevated FIB-4, only a limited number was referred for elastography (2/188, 1.1%) or to hepatology (3/188, 1.6%) (Figure 1). In addition, patients with abnormal liver chemistry also had lower rates for elastography ordered by their primary care provider (5/521; 1.0%).

## DISCUSSION

This proof-of-concept study provides a novel approach to automating detection and risk stratification of patients with NAFLD through EHR functionality in a prospective cohort of more than 2100 patients. The absence of necessary laboratory testing for FIB-4 assessment of NAFLD can limit FIB-4 use in practice and is a significant care gap that can be easily identified electronically. In addition, this approach detected significant care gaps among patients with NAFLD and available laboratory testing in that only ~3% of patients with abnormal FIB-4 were referred for either elastography or hepatology consultation. Conversely, 2% of patients without laboratory testing were referred to hepatology or elastography. If labs had been obtained and were normal, many of these patients could have been saved a hepatology referral or an elastography. This approach can potentially provide remarkable and sustainable support to primary care providers in the assessment of patients with NAFLD, optimize resource utilization, and permit early detection of NAFLD when it is at reversible stages.

Importantly, this approach introduces a feasible clinical decision support system for widespread implementation given the capability to share functionality between sites on the same EHR. The implications of this approach are numerous. Utilizing this algorithm as a clinical decision support system could alert primary care providers about the need for linkage to NAFLD care. Future implementations should quantify the algorithm’s performance characteristics in clinical practice. Patients with low FIB-4 could be potentially saved from costly imaging studies or unnecessary referrals, thereby directing this needed subspecialty care toward patients with advanced disease. Prompting early linkage of patients with elevated FIB-4 or elevated liver enzymes to elastography-based assessment or hepatology care, respectively, will certainly increase the rate of detection of advanced fibrosis and cirrhosis. Such detection is instrumental to the life-saving early detection of HCC through linkage to screening programs and for disease reversal, when possible, through active weight loss-based interventions. The current limitations for this clinical decision support system relate to NAFLD detection through reliance on diagnostic codes, which can potentially be addressed by the future utilization of natural language processing, machine learning, or embedding additional algorithmic detection schemes within the EHR.^10^

The results of this initiative raise important questions regarding capacity for the potential massive influx of patients to hepatology clinics that could occur secondary to implementation of any effective tool for identification of patients with advanced stages of NAFLD in primary care settings. Center limitations may prohibit timely completion of elastography if ordering frequency among patients with elevated FIB-4 were to rise dramatically. Further studies are imperative to directly assess the magnitude of impact (or the consequent strain) of CDS tools at the level of patient outcomes, providers, and health care systems. In summary, our results support the capability of utilizing EHR functionality for a novel approach to detect NAFLD-related care gaps at both individual and systematic levels and can consequently optimize linkage to care and resource utilization.
